# Supplementary material for: The impact of outpatient settlement for cross-regional medical treatment on healthcare choice by the floating population: PSM+DID evidence based on CFPS
Source: PLoS One. 2025 May 8;20(5):e0323127. doi: 10.1371/journal.pone.0323127 (PMC12061399; doi:10.1371/journal.pone.0323127)
Supplement: Attachment — Submitted filename: Response to Reviewers.docx [file pone.0323127.s002.docx]

**Response to Reviewers**

**Date:** January 15, 2025

**Manuscript Title:** The impact of outpatient settlement for cross-regional medical treatment on healthcare choice by the floating population: PSM+DID evidence based on CFPS

**Manuscript ID:** PONE-D-24-36231

Dear Dr. Matteo and reviewers,

We sincerely appreciate your recognition of our study titled “The impact of outpatient settlement for cross-regional medical treatment on healthcare choice by the floating population: PSM+DID evidence based on CFPS” (ID: PONE-D-24-36231) and the comments you provided. Your insights have significantly contributed to improving our manuscript, and we are deeply grateful for the time and effort you dedicated to reviewing our work. We have carefully reviewed each of your comments and have thoroughly revised the manuscript, clarifying unclear points, refining the policy background for better reader comprehension, and clarifying the contribution of our study to an international audience. Below, we provide the original comments and our responses, with our responses marked in italics for clarity.

**Response to Dr. Matteo**

1. **Comment:** Please ensure that your manuscript meets PLOS ONE's style requirements, including those for file naming. The PLOS ONE style templates can be found at https://journals.plos.org/plosone/s/file?id=wjVg/PLOSOne_formatting_sample_main_body.pdf and <https://journals.plos.org/plosone/s/file?id=ba62/PLOSOne_formatting_sample_title_authors_affiliations.pdf>

***Response:*** *We appreciate your careful review of the manuscript's formatting. We have revised and corrected the title capitalization, author addresses, and the font and size throughout the manuscript in accordance with the PLOS ONE’s style requirements.* *We hope that the new version of the manuscript is now in total accord with the Journal formatting requirements.*

2. **Comment:** Please amend either the title on the online submission form (via Edit Submission) or the title in the manuscript so that they are identical.

***Response:*** *We have carefully verified both the title in the online submission and the title in the manuscript, making revisions to ensure they are identical.*

3. **Comment:** Please match your authorship list in your manuscript file and in the system.

***Response:*** *Following your suggestion,* *we have matched the authorship list in the manuscript with the one in the system.* *Additionally, due to Dr. Dawei Zhang's contributions during the response to reviewers stage, he will be added as a co-corresponding author, and we have made revisions to the author order and corresponding authors information.*

***Original:***

*“Qiang Su, Yumeng Zhang, Yuanhao Sui, Dawei Zhang and Lihua Sun**

*Department of Pharmacy Administration, School of Business Administration, Shenyang Pharmaceutical University, 103 Wenhua Road, Shenyang, 110016, Liaoning Province, PR China*

** Corresponding author*

*E-mail: slh-3632@163.com(SLH)”*

***Revised:***

*“Qiang Su^1^, Yumeng Zhang^1^, Yuanhao Sui^1^, Lihua Sun^1,*^, Dawei Zhang^1,*^*

*1 Department of Pharmacy Administration, School of Business Administration, Shenyang Pharmaceutical University, Shenyang, Liaoning Province, China*

*******	*Corresponding authors*

*E-mail:* [*slh-3632@163.com (LS)*](mailto:slh-3632@163.com%20(LS))*; nkelite@163.com (DZ)”*

4. **Comment:** Please include a caption for figure 1, 2 and 5.

***Response:*** *We sincerely appreciate your thorough review and apologize for any inconvenience caused. After a careful check, we found that the original manuscript already included the figure titles for figures 1, 2, and 5. In the Revised Manuscript with Track Changes, we have highlighted all figure titles in yellow (on lines 261, 275, 276, 290, and 300, respectively). The figure titles in the manuscript are as follows:*

***Fig 1.*** ***Common support assumption.***

***Fig 2. Propensity score kernel density plot before the match.***

***Fig 3. Propensity score kernel density plot after the match.***

***Fig 4. Parallel trends test.***

***Fig 5. Placebo test.***

5. **Comment:** PLOS requires an ORCID iD for the corresponding author in Editorial Manager on papers submitted after December 6th, 2016. Please ensure that you have an ORCID iD and that it is validated in Editorial Manager. To do this, go to ‘Update my Information’ (in the upper left-hand corner of the main menu), and click on the Fetch/Validate link next to the ORCID field. This will take you to the ORCID site and allow you to create a new iD or authenticate a pre-existing iD in Editorial Manager.

***Response:*** *Thank you very much for your reminder. The corresponding author, Lihua Sun, has linked the ORCID (0009-0001-1705-6313).*

**Response to Reviewer #1**

1. **Comment:** This paper investigates the impact of cross-regional outpatient reimbursement policies on the medical treatment choices of floating populations in China using a combination of Propensity Score Matching (PSM) and Difference-in-Differences (DID) methodologies. The results provide empirical evidence that these reimbursement policies significantly encourage floating individuals to seek care at higher-tier medical institutions, which may be at odds with the goals of China’s hierarchical medical system. The study uses a robust dataset (China Family Panel Studies, CFPS) and presents thorough analysis techniques.

***Response:*** *We sincerely appreciate the time and effort you dedicated to thoroughly reviewing our manuscript and accurately summarizing the overall content of our study. We are truly honored to have received your recognition of our manuscript.*

2. **Comment:** Clarity and language: The manuscript is generally well-organized and follows a logical structure. However, there are a few areas where the paper could benefit from more clarity (e.g., grammar issues, conciseness) or elaboration, especially in the abstract and introduction. For example, the phrasing of the second sentence in the abstract “This study seeks to investigate the influence of outpatient reimbursement for cross-regional medical services on floating individual medical treatment decision” could be reworded as: “This study examines how outpatient reimbursement for cross-regional medical services influences the medical treatment decisions of floating individuals.” Again, “Empirical analysis suggest that floating population are more likely…” (line 25) should be “Empirical analysis suggests that the floating population is more likely…”. “Cross-regional outpatient reimbursement policy effect floating population” (line 29): this should be “affect” instead of “effect.” More generally, the use of terms like “floating population” and “high-tier medical institutions” are somewhat vague and could benefit from more precise definitions early in the paper to avoid ambiguity for readers less familiar with Chinese healthcare policy. Overall, I suggest having the paper proofread by an English native speaker before further submitting it.

***Response:*** *We are grateful for your recognition of our manuscript's structure and logical consistency, as well as for your thoughtful and detailed revision suggestions. We have thoroughly addressed the grammatical issues and made improvements to the areas where the expressions were not concise or clear:*

*1) To ensure that readers unfamiliar with Chinese healthcare policies can more easily understand the content, we have refined the language and thoroughly reviewed the entire manuscript. We replaced “high-tier medical institutions” with “advanced medical institution” and added an explanation the first time “floating population” appears in the manuscript, specifying that it refers to a population “…who live and work in regions different from their registered residence.” (on lines 24-25 of the manuscript and lines 27-28 of the revised manuscript with track changes.)*

*2)* *According to your suggestion, the phrasing of the second sentence in the abstract, “This study seeks to investigate the influence of outpatient reimbursement for cross-regional medical services on floating individual medical treatment decision” has been reworded as: “This study examines the impact of outpatient settlement for cross-regional medical treatment on healthcare choices by the floating population...” (on lines 23-24 of the manuscript and lines 25-27 of the revised manuscript with track changes.)*

*3) The sixth sentence of the abstract, “Empirical analysis suggest that floating population are more likely…” has been reworded as: “The findings indicate that they are significantly more inclined to select…”(on lines 27-28 of the manuscript and lines 32-33 of the revised manuscript with track changes.)*

*4)* *The “effect” in the seventh sentence of the abstract has been revised to “affect”. (on line 29 of the manuscript and line 35 of the revised manuscript with track changes.)*

*5) It is a great suggestion to have an English native speaker proofread the manuscript before submission. The manuscript has been proofread by an English native speaker, and the revisions have been marked in the file labeled 'Revised Manuscript with Track Changes' using the Word revision mode.*

3. **Comment:** Outpatient reimbursement reform for cross-regional medical services: The explanation of the outpatient reimbursement reform in the study could benefit from much more clarity. Specifically, they mention the introduction of a cross-regional reimbursement system for outpatient services as part of China’s healthcare reform, but there’s a lack of detailed explanation about how this policy is structured and implemented in real-world terms. For a better understanding, several aspects of the reform could have been elaborated further, such as:

- Policy Mechanism: While the study mentions that the reimbursement for cross-regional medical services was initially focused on hospitalization and later expanded to outpatient services, it doesn’t explain the process in detail. How do individuals access care in different regions, and what steps are involved in the reimbursement process? How do patients know if they are eligible for reimbursement or how to apply for it?

- Pilot Areas: The authors refer to the pilot regions where the reimbursement system was first introduced, but do not explain how these geographical areas were chosen. Do these regions have specific geographical or demographic characteristics? Are they more urban or rural? Did these characteristics play a role in the selection process?

- Coordination with Hierarchical Medical System: The authors argue that the policy conflicts with the goals of the hierarchical medical system, but they do not fully explain how the hierarchical system is supposed to work in the first place. A clearer explanation of this system, and why the cross-regional reimbursement reform disrupts it, would help readers better understand the policy's implications (see also comment below).

***Response:*** *We sincerely thank you for your valuable comments. Your thoughtful comments have provided clear guidance on areas of the manuscript that require further improvement. We have made modifications and additions to the introduction, mainly focusing on the policy mechanisms, the selection of pilot regions, and the coordination with the hierarchical medical system. The detailed revisions are as follows:*

*1) Policy Mechanism:*

*We have supplemented the revised manuscript with relevant content, systematically reviewing the reform trajectory of the cross-regional medical treatment policy from hospitalization to outpatient reimbursement, and providing a detailed explanation of the specific process for outpatient reimbursement. We have provided a detailed explanation of the policy development process in the first paragraph of the introduction: “As early as March 2009, the Opinions of the Central Committee of the Communist Party of China and the State Council on Deepening the Reform of the Medical and Healthcare System [4] proposed prioritizing the transfer and continuation of basic medical insurance for rural migrant workers and improving settlement services for retirees receiving medical care cross-regional. This initiative launched direct settlement services for cross-regional medical expenses, initially focusing on hospitalization costs [5]. Following the establishment of the National Healthcare Security Administration (NHSA), the scope of cross-regional medical reform has been further expanded. In May 2018, the NHSA launched cross-regional direct settlement for outpatient expenses [6]. By September of the same year, the Yangtze River Delta region (Shanghai, Jiangsu, Zhejiang, and Anhui) was selected as the first pilot region due to its high population mobility and pressing demand for cross-regional medical treatment [7]. In December 2019, the southwest region (Sichuan, Chongqing, Guizhou, Yunnan, and Xizang) and the Beijing-Tianjin-Hebei region also initiated pilot programs for outpatient expense settlement [8]. These pilots were designed to test the policy’s feasibility in regions with varying economic development levels and healthcare resource distributions. On the basis of the initial success of these pilots, in September 2020, the NHSA and the Ministry of Finance jointly issued the Notice on the Issuance of the List of Newly Added Pilot Provinces for Cross-Regional Direct Settlement of Outpatient Expenses, signaling the nationwide implementation of cross-regional direct settlement for outpatient expenses [9].” (on lines 44-60 of the manuscript and lines 51-67 of the revised manuscript with track changes.) We have provided a detailed explanation of the reimbursement conditions and process in the second paragraph of the introduction: “Patients seeking medical treatment cross-regional can complete the required pre-treatment filing procedures in advance through various online and offline channels, including the National Medical Insurance Service Platform app, the mini-program on the State Council mobile for cross-regional medical services, or the service windows of insurance agencies. Once the filing is completed, patients are able to access medical services at designated medical institutions using valid credentials such as the electronic medical insurance certificate or social security card. When settling medical expenses, individuals only need to pay the portion they are personally responsible for, without the need to advance payments or go through additional procedures to receive medical insurance reimbursement [10].” (on lines 63-71 of the manuscript and lines 70-78 of the revised manuscript with track changes.)*

*2)* *Pilot Areas*

*We have provided a detailed supplement in the revised manuscript regarding the selection criteria for pilot regions and their characteristics: “By September of the same year, the Yangtze River Delta region (Shanghai, Jiangsu, Zhejiang, and Anhui) was selected as the first pilot region due to its high population mobility and pressing demand for cross-regional medical treatment [7]. In December 2019, the southwest region (Sichuan, Chongqing, Guizhou, Yunnan, and Xizang) and the Beijing-Tianjin-Hebei region also initiated pilot programs for outpatient expense settlement [8]. These pilots were designed to test the policy’s feasibility in regions with varying economic development levels and healthcare resource distributions. On the basis of the initial success of these pilots, in September 2020, the NHSA and the Ministry of Finance jointly issued the Notice on the Issuance of the List of Newly Added Pilot Provinces for Cross-Regional Direct Settlement of Outpatient Expenses, signaling the nationwide implementation of cross-regional direct settlement for outpatient expenses [9].” (on lines 51-60 of the manuscript and lines 58-67 of the revised manuscript with track changes.)*

*3)* *Coordination with Hierarchical Medical System*

*In the third paragraph of the introduction, we have provided a detailed description of the goals and implementation methods of the hierarchical medical system while also explaining the reasons why outpatient settlement for cross-regional medical treatment impacts this system: “The primary objective of the hierarchical medical system is to optimize the allocation of medical resources by promoting the division of labor and cooperation among various levels of medical institutions. This aims to address the issue of overcrowding in advanced medical institutions and underutilization of resources in primary healthcare facilities, ultimately achieving a healthcare delivery model where patients with minor illnesses are treated in community-level institutions, serious illnesses are treated in general hospitals and recovery occurs in the community. Nevertheless, the increasing accessibility of outpatient settlement for cross-regional medical treatment would motivate a part of insured individuals to seek treatment at advanced medical institutions outside their registered residence. This trend could lead to a rise in outpatient visits to these advanced medical institutions, thereby hindering the implementation of the hierarchical medical system.” (on lines 74-82 of the manuscript and lines 81-89 of the revised manuscript with track changes.)*

4. **Comment:** Literature review: The literature review section highlights the significance of cross-regional medical services in China and points out that there is limited research on the effect of outpatient reimbursement policies on individual treatment choices. The references used seem relevant, but the author could expand on the findings and limitations of existing studies more explicitly, which could provide stronger justification for the present study.

***Response:*** *Thank you for your thoughtful review and for recognizing the citation section of the literature review in our manuscript. Following your comments, we have further expanded the existing literature, summarized its limitations, and provided a stronger justification for our study. We have provided a detailed presentation in the fourth paragraph of the introduction: “Cross-regional medical treatment has become prevalent in Europe and the United States. Due to differences in healthcare systems abroad compared to China, such practices are generally referred to as “medical tourism” or “cross-border healthcare” in international contexts [11,12]. International scholars primarily focus on two aspects of Cross-regional medical treatment in their research. The first aspect is the driving factors of cross-regional medical treatment. Scholars have noted that it is driven by factors such as the pursuit of life extension, distrust of regional doctors, and the demand for more convenient and higher-quality medical services [13–17]. The second aspect is the impact of cross-regional medical treatment. Findings indicate that, due to convenience and quality, patients tend to utilize more healthcare resources and prefer advanced medical institutions to primary, which ultimately results in elevated medical costs [18–20]. Compared with international research, Chinese scholars have predominantly concentrated on the benefits of outpatient settlement for cross-regional medical treatment. Studies [21–23] have demonstrated that outpatient settlement for cross-regional medical treatment enhances the convenience of flotation individuals, promoting public welfare. Additionally, Zhong Yuyin [24] analyzed settlement data for medical insurance before and after the implementation of outpatient settlement for cross-regional medical treatment in pilot cities, revealing that this policy led to an increase in patient visits to advanced medical institutions, which poses challenges to the effective implementation of the hierarchical medical system. Similarly, using hospital data, Yang Xi and her team [25] found that the average cost per visit for patients utilizing cross-regional settlement was approximately 5,000 Chinese Yuan (CNY) higher than that of locally insured patients. Existing studies primarily focus on cities and hospitals as research subjects to examine the impact of outpatient settlement for cross-regional medical treatment. However, there is a lack of research exploring the influence of this policy on individuals' healthcare choices." (on lines 84-103 of the manuscript and lines 99-111 of the revised manuscript with track changes.)*

5. **Comment:** Methods: The methodology section is clearly articulated, with detailed explanations of the data source (CFPS) and statistical techniques (PSM+DID). The authors have justified their choice of methods, citing relevant studies. The use of PSM to account for selection bias and DID to examine policy effects is appropriate for this type of analysis.

• Sample Size: The sample size (3,019 individuals) is sufficient for the analysis, but the authors should explain how the sample was chosen more explicitly. Was there any weighting or criteria in the sample selection process? This should be clarified.

• Variable Description: The explanation of variables (e.g., medical choice, treatment, income, etc.) is mostly clear. However, “Medical choice” as the dependent variable could use a more detailed description. How exactly is this variable measured? It would help the reader to understand its operationalization in the context of this study. Furthermore, the statement “To assess the influence of the outpatient reimbursement policy...” could be rewritten for clarity. Instead of “variables such as 'treatment' (treat), 'policy time' (time), and their product double difference term (did),” you could say: “Key independent variables include 'treatment' (indicating whether the individual sought treatment), 'policy time' (indicating the implementation period of the policy), and the interaction term (did) that captures the policy’s effect.”

• Model Specification: The use of the multi-period DID method is a strength, as it accounts for varying policy implementation times across provinces. However, more detail could be provided regarding the reasoning for the specific model used and the control variables. For instance, why were certain covariates like “rust in doctors” and “health status” chosen, and how might these influence treatment decisions?

***Response:*** *Thank you for your detailed review and valuable comments on the methods section of our manuscript. We are honored by your recognition of our choice to use CFPS data and the application of the PSM+DID method to account for selection bias and examine policy effects, as well as your recognition of the clarity of the explanation. Your comments have been instrumental in helping us further refine the manuscript, enhancing its clarity and rigor. Below are our responses to the specific issues you raised:*

*1)* *Sample Size:* *We have added the process and criteria for sample selection in the “Data Sources” section of the manuscript: “The data for this research were sourced from 2012 to 2020. The data were screened based on the study subjects and the logic of the survey questionnaire, applying the following inclusion and exclusion criteria, a final sample size of 3,019 individuals was included: 1) Individuals with missing household registration information for any year were excluded; 2) Individuals whose residence and household registration information were the same at any year were excluded; 3) Only those individuals whose residence and household registration were both located within mainland China were included.” (on lines 114-119 of the manuscript and lines 142-147 of the revised manuscript with track changes.)*

*2)* *Variable Description:* *We have added “Table 1.* *Meaning of variables.” in the “Variables” section of the manuscript, which provides a detailed description of the definition and assignment of variables. (on lines 135-136 of the manuscript and lines 171-172 of the revised manuscript with track changes.) “To assess the influence of the outpatient reimbursement policy...” have been revised to “This study assesses the impact of outpatient reimbursement for...” (on line 121 of the manuscript and line 151 of the revised manuscript with track changes.) “variables such as ‘treatment’ (treat), ‘policy time’ (time), and their product double difference term (did) were derived.” have been revised to “*key independent variables include ‘treat’ (indicate whether the policy was implemented in the region), ‘time’ (indicate whether the policy was implemented at that time), and the interaction term ‘did’ (capturing the policy’s effect).*” (on lines 124-126 of the manuscript and lines 155-157 of the revised manuscript with track changes.) “variables such as personal annual income (shouru), medical institution level (hl), degree of trust in doctors (bod), and personal health status (soh), which affect the treatment variable (treat), are employed as covariates for this purpose.” have been revised to “*variables such as “shouru” (personal annual income), “hl” (level of the medical institution where treatment is sought), “bod”(degree of trust in doctors), and “soh”(personal health status), which affect the treatment variable “treat”, are chosen as covariates for this analysis.*” (on lines 128-130 of the manuscript and lines 160-164 of the revised manuscript with track changes.) “this study uses these factors as control variables, including age (age), intelligence level (itlg), and hospitalization in the past year (hpl), among others.” have been revised to “*this study includes these factors as control variables, such as “age”, “itlg”(intelligence level), and “hpl”(hospitalization in the past year), among others.*” (on lines 132-134 of the manuscript and lines 167-169 of the revised manuscript with track changes.)*

*3)* *Model Specification: Thank you for your professional and thorough review. We are very pleased to receive your recognition. When the treatment times differ among individuals in the treatment group, applying a multi-period DID is appropriate. We have explained the rationale for using multi-period DID in the second paragraph of the "Statistical Methods" section of the manuscript:"* *Traditional DID analysis necessitates that policy implementation times remain consistent among individuals in the experimental group. However, the policy of outpatient reimbursement for cross-regional medical treatment is introduced on a provincial basis, resulting in varying implementation times for individuals in the experimental group. Consequently, this study utilizes multi-period DID [33] to assess the differences before and after intervention between the experimental and control groups, both of which implement the policy at different time points." (on lines 142-147 of the manuscript and lines 181-187 of the revised manuscript with track changes.) The selection of control variables is crucial. We have considered variables that previous literature has shown to influence medical choices,* *combined with practical considerations from the CFPS dataset. The final set of control variables used in our study is explained in the last paragraph of the "Variables" section of the manuscript:"* *Previous research [28–32] has demonstrated that various factors influence an individual's healthcare choice. Therefore, drawing on prior studies and practical considerations from the CFPS dataset, this study includes these factors as control variables, such as “age”, “itlg” (intelligence level), and “hpl” (hospitalization in the past year), among others.” (on lines 131-134 of the manuscript and lines 165-169 of the revised manuscript with track changes.)*

6. **Comment:** The Impact of the Reform on Different Groups: Although the study finds that certain groups (urban residents, employed individuals) are more affected by the reform, there’s not enough context about why this might be the case. What features of the reform make it particularly impactful for these groups? Is it due to their access to information, ability to navigate the system, or perhaps their greater reliance on higher-level medical institutions in urban settings?

***Response:*** *We appreciate your insightful suggestions, which have contributed significantly to the improvement of our manuscript. In the discussion section, we have provided a detailed explanation of the reasons behind the differential impact of the policy on various groups and, based on these findings, have proposed recommendations for improvement: “This disparity arises from the limitations inherent in the policy pilot. Urban residents and employees are more likely to access information about the policy and to select advanced medical institutions nearby, while rural residents encounter challenges due to transportation issues and information asymmetry. To enhance policy implementation and promote equity, it is essential to gradually expand the policy coverage to encompass more grassroots medical institutions within the reimbursement system. Additionally, providing clear guidance through village committees and primary medical institutions can assist rural residents in understanding and actively engaging with the policy.” (on lines 281-284 of the manuscript and lines 343-346 of the revised manuscript with track changes.)*

7. **Comment:** Policy implications: While both the discussion and conclusion sections briefly touch on policy recommendations, they could delve deeper into the policy implications of the findings. Specifically, while the paper’s findings show that cross-regional reimbursement encourages higher-tier institution use, the authors do not fully address how the policy could be improved to align better with the goals of the hierarchical medical system (e.g., promoting more equitable access, and ensuring efficient resource allocation). A deeper analysis of potential trade-offs and policy levers could be beneficial.

***Response:*** *A deeper analysis of potential trade-offs and policy levers has indeed been of great help in enhancing our manuscript. We appreciate your suggestions. In the discussion section, we have provided specific recommendations on how to balance public fairness and convenience with the efficient allocation of medical resources, as well as ensure alignment between the cross-regional outpatient reimbursement policy and the tiered healthcare system: “In policy formulation and implementation, the pharmaceutical, medical insurance, and healthcare sectors must balance public fairness and convenience with the efficient allocation of medical resources. To facilitate this, advanced medical institutions should be encouraged to support primary healthcare institutions by deploying experts to grassroots healthcare and leveraging telemedicine. These approaches would enhance the capabilities of primary healthcare institutions and foster collaborative mechanisms for resource sharing. The reimbursement process should also be refined, and ratios should be adjusted to promote organized medical treatment. This would reduce unnecessary visits to advanced medical institutions while ensuring that the outpatient reimbursement for cross-regional medical treatment policy aligns effectively with the hierarchical medical system.” (on lines 287-295 of the manuscript and lines 350-358 of the revised manuscript with track changes.)*

**Response to Reviewer #2**

1. **Comment:** The authors present a paper about the impact of cross-regional outpatient reimbursement policies on the medical treatment decisions of the floating population in China. Using data from the China Family Panel Studies (CFPS) spanning 2012 to 2020, the authors adopt Propensity Score Matching and Difference-in-difference methodologies for their empirical analysis. The study highlights policy-induced shifts in healthcare-seeking behavior, revealing mismatches between policy objectives and outcomes. It suggests a need for better coordination among healthcare policies, improved infrastructure in under-resourced areas, and adjustments to ensure alignment with hierarchical system goals. The findings offer insights for policymakers aiming to refine reimbursement policies and address healthcare inequities in China.

***Response:*** *Thank you for your thorough review and valuable comments on our manuscript. We are pleased that you provided a comprehensive summary of our study and offered positive comments on the topic and research methodology.*

2. **Comment:** Over all the text there are several points, starting from abstract and introduction, where a blank space between words is missing.

***Response:*** *We greatly appreciate your thorough review. We have carefully checked all the sentences in the manuscript and have it proofread by an English native speaker.*

3. **Comment:** In page 7 Table 3 is indicated as Table 2.

***Response:*** *Thank you for your reminder, and we apologize for any confusion caused. After careful examination and attempts, we found no issue with the content of “Table 3” on page 7 of the submitted manuscript. However, the PDF generated by the submission system always converts “Table 3” into “Table 2”. We reported this issue to the journal when we submitted the manuscript for the first time and received a patient response from them. Ultimately, following the journal's suggestion, we have uploaded a separate file clarifying the title of “Table 3”, which has resolved the issue.*

4. **Comment:** In the introduction the authors affirm that “According to the most recent national census data from China, there is a notable phenomenon of household separation. This has exacerbated disparities in the distribution of medical resources and insurance benefits across various regions”. This sentence is generic, and I do not understand what it means. I think that a more specific explanation is needed.

***Response:*** *Thank you for your valuable comments on the introduction section. We sincerely apologize for any confusion and have made further additions and specific explanations to articulate the background and logic of this issue more clearly: “According to the latest Chinese national population census, there has been an observable increase in population mobility, accompanied by an inconsistency between household registration and actual residential locations [1]. This phenomenon has led to an increasing misalignment between individuals’ health insurance enrollment locations and their actual places of residence [2]. In particular, with the increasing demand for high-quality medical resources and the concentration of inter-regional migrants in economically developed regions [3], this misalignment results in the underutilization of healthcare resources and a decrease in the overall efficiency of medical resource allocation. Such challenges exacerbate disparities in healthcare resource distribution and social health insurance benefits inequalities.” (on lines 36-43 of the manuscript and lines 43-50 of the revised manuscript with track changes.)*

5. **Comment:** In the introduction some papers on cross-regional patients’ migration are discussed, but they seem focused only in China. I think that Plos One is devoted to an international panel of researchers and then the state of art and the rest of the paper should be focused to provide a contribution for a most extended audience.

***Response:*** *Thank you for your constructive comments. Following your suggestion, we broadened the scope of the literature review by extensively reviewing and summarizing international literature on cross-regional medical treatment, and we have cited relevant studies published in international journals, including PLOS ONE. The specific content is as follows: “Cross-regional medical treatment has become prevalent in Europe and the United States. Due to differences in healthcare systems abroad compared to China, such practices are generally referred to as “medical tourism” or “cross-border healthcare” in international contexts [11,12]. International scholars primarily focus on two aspects of Cross-regional medical treatment in their research. The first aspect is the driving factors of cross-regional medical treatment. Scholars have noted that it is driven by factors such as the pursuit of life extension, distrust of regional doctors, and the demand for more convenient and higher-quality medical services [13–17]. The second aspect is the impact of cross-regional medical treatment. Findings indicate that, due to convenience and quality, patients tend to utilize more healthcare resources and prefer advanced medical institutions to primary, which ultimately results in elevated medical costs [18–20].” (on lines 84-92 of the manuscript and lines 91-99 of the revised manuscript with track changes.)*

6. **Comment:** For the data analysis China Family Panel Studies is used, but it is not clear how the authors select 3,019 individuals. They state that this is a sample, but I do not understand if it is a representative sample or not, if they applied any selection criteria etc. All this part needs to be clarified.

***Response:*** *Thank you for your thorough review. We have added the process and criteria for sample selection in the “Data Sources” section of the manuscript: “The data for this research were sourced from 2012 to 2020. The data were screened based on the study subjects and the logic of the survey questionnaire, applying the following inclusion and exclusion criteria, a final sample size of 3,019 individuals was included: 1) Individuals with missing household registration information for any year were excluded; 2) Individuals whose residence and household registration information were the same at any year were excluded; 3) Only those individuals whose residence and household registration were both located within mainland China were included.” (on lines 114-119 of the manuscript and lines 142-147 of the revised manuscript with track changes.)*

7. **Comment:** The dependent variable hq is measured on a scale between 1 to 5 (see Table 1), but the equation (1) seems to be estimated using an OLS approach. Even in this case I do not understand how the authors approach the estimation process.

***Response:*** *Thank you for your valuable insights, and we apologize for any confusion caused. As you correctly pointed out, the dependent variable hq is an ordered variable ranging from 1 to 5. We understand your concerns, and indeed, we had considered using GLM models such as logistic or probit for estimation. However, after careful consideration, we decided to use OLS, which is more widely applied in DID and yields results that are easier for readers to understand. Furthermore, since the dependent variable hq is ordinal, we felt that OLS would still be an appropriate choice. To clarify the estimation process, we have added the phrase “Estimates of DID are obtained using the Ordinary Least Squares (OLS) method” at the end of the second paragraph in the "Statistical Methods" section of the manuscript. (on line 150 of the manuscript and lines 190-191 of the revised manuscript with track changes.) Once again, thank you for your thoughtful comment. It has provided important insights for our future research. In future studies, we will further explore how to use more suitable models while presenting results that are easy to interpret.* *We have provided a detailed explanation of the study's limitations in the last paragraph of the Discussion section of the manuscript:“ Of course, there are certain limitations to this study. To facilitate readers' comprehension, the DID regression in this study was conducted using the widely adopted OLS method. Future research could consider employing various methods simultaneously, such as Generalized Linear Models (GLM) and Machine Learning (ML) methods, for DID regression. On this basis, the optimal model can be selected and could explore more comprehensible interpretations of the results for the optimal model.” (on lines 296-300 of the manuscript and lines 362-366 of the revised manuscript with track changes.)*

8. **Comment:** In Table 2, where the regression coefficients are presented, N is equal to 2,728, a reduced number compared to the overall 3,019. Is this a consequence of the PSM application? In addition, the authors cite the paper number 24 and they say that “matching effect is optimal when the number of neighbors in the propensity score matching method is four”. Does this mean that in the 2,728 selected observations we have a ratio of 1:4 between treated and untreated?

***Response:*** *Thank you for your professional and detailed review. We completely agree with your point. In Table 2 (now Table 3), the sample size for the regression is 2,728, which is smaller than the total sample size of 3,019. This reduction is because after performing propensity score matching (PSM), only the matched samples (those with nonzero weights) were used in the DID regression. This is also explained in the footnote of Table 3: “Both regressions (1) and (2) use data with nonzero weights after propensity score matching.” (on line 187 of the manuscript and line 232 of the revised manuscript with track changes.) Referring to reference [34] Abadie A, Drukker D, Herr JL, Imbens GW. Implementing matching estimators for average treatment effects in Stata. Stata J. 2004;4: 290–311. doi:10.1177/1536867X0400400307, the number of neighbors for PSM was set to 4, meaning that for each individual in the treatment group, 4 of the most similar individuals from the control group were selected for matching. In the DID regression, only the matched samples were used. As a result, 4 control group individuals matched to each treatment group individual may overlap, meaning the ratio of sample sizes between the treatment and control groups is not necessarily 1:4. Upon reviewing the original data, the number of matched individuals in the treatment and control groups was found to be 1,357 and 1,371, which gives a ratio close to 1:1. This has also been noted in the footnote of Table 3:* *“The treatment and control groups consist of 1,357 and 1,371.” (on lines 187-188 of the manuscript and lines 232-233 of the revised manuscript with track changes.)*

9. **Comment:** I would like to see a Table of descriptive statistics where the main variables considered are split between treated and control units.

***Response:*** *This is an excellent suggestion that helps us further improve the presentation of the manuscript.* *We have revised “Table 2. Descriptive Statistics of Variables” (on line 178 of the manuscript and line 222 of the revised manuscript with track changes.) by including the variables “treat,” “time,” and “did” in the original table. Additionally, we have added “Table 1. Meaning of Variables” (on line 135 of the manuscript and line 171 of the revised manuscript with track changes.) offers a detailed description of each variable's definition and assignment. The variables are now categorized into four groups: (1) Dependent Variable, (2) Key Independent Variables, (3) Control Variable, and (4) Covariates for PSM.*

10. **Comment:** It is not clear in the conclusion the contribution of the paper to an international audience.

***Response:*** *Thank you for the insights you have provided. We have added content in the conclusion section to explain the contribution of this study to the international audience: “Moreover, the findings contribute insights for enhancing such policies in countries with similar floating population and hierarchical medical system, and they also furnish pertinent information for countries that have yet to implement such policies.” (on lines 309-311 of the manuscript and lines 376-379 of the revised manuscript with track changes.)*

11. **Comment:** A brief explanation of the healthcare system should be included.

***Response:*** *Thank you for your comments. We have further expanded on the relevant content regarding outpatient settlement for cross-regional medical treatment in the introduction, including its background, development history, reimbursement conditions and processes, as well as its impact on the tiered healthcare system and reasons: “According to the latest Chinese national population census, there has been an observable increase in population mobility, accompanied by an inconsistency between household registration and actual residential locations [1]. This phenomenon has led to an increasing misalignment between individuals’ health insurance enrollment locations and their actual places of residence [2]. In particular, with the increasing demand for high-quality medical resources and the concentration of inter-regional migrants in economically developed regions [3], this misalignment results in the underutilization of healthcare resources and a decrease in the overall efficiency of medical resource allocation. Such challenges exacerbate disparities in healthcare resource distribution and social health insurance benefits inequalities. Consequently, cross-regional medical treatment has become a focal point in healthcare reform efforts [2]. As early as March 2009, the Opinions of the Central Committee of the Communist Party of China and the State Council on Deepening the Reform of the Medical and Healthcare System [4] proposed prioritizing the transfer and continuation of basic medical insurance for rural migrant workers and improving settlement services for retirees receiving medical care cross-regional. This initiative launched direct settlement services for cross-regional medical expenses, initially focusing on hospitalization costs [5]. Following the establishment of the National Healthcare Security Administration (NHSA), the scope of cross-regional medical reform has been further expanded. In May 2018, the NHSA launched cross-regional direct settlement for outpatient expenses [6]. By September of the same year, the Yangtze River Delta region (Shanghai, Jiangsu, Zhejiang, and Anhui) was selected as the first pilot region due to its high population mobility and pressing demand for cross-regional medical treatment [7]. In December 2019, the southwest region (Sichuan, Chongqing, Guizhou, Yunnan, and Xizang) and the Beijing-Tianjin-Hebei region also initiated pilot programs for outpatient expense settlement [8]. These pilots were designed to test the policy’s feasibility in regions with varying economic development levels and healthcare resource distributions. On the basis of the initial success of these pilots, in September 2020, the NHSA and the Ministry of Finance jointly issued the Notice on the Issuance of the List of Newly Added Pilot Provinces for Cross-Regional Direct Settlement of Outpatient Expenses, signaling the nationwide implementation of cross-regional direct settlement for outpatient expenses [9].*

*The direct settlement of cross-regional medical expenses has established a unified network for cross-regional medical insurance settlements, enabling interconnectivity among different medical insurance pooling areas and overcoming the limitations of the traditional localized management system. Patients seeking medical treatment cross-regional can complete the required pre-treatment filing procedures in advance through various online and offline channels, including the National Medical Insurance Service Platform app, the mini-program on the State Council mobile for cross-regional medical services, or the service windows of insurance agencies. Once the filing is completed, patients are able to access medical services at designated medical institutions using valid credentials such as the electronic medical insurance certificate or social security card. When settling medical expenses, individuals only need to pay the portion they are personally responsible for, without the need to advance payments or go through additional procedures to receive medical insurance reimbursement [10]. This measure has significantly simplified the reimbursement process for cross-regional medical insurance patients, enhancing the convenience and efficiency of medical insurance services. However, the increased convenience may also lead to complex interactions and impacts on the hierarchical medical system.*

*The primary objective of the hierarchical medical system is to optimize the allocation of medical resources by promoting the division of labor and cooperation among various levels of medical institutions. This aims to address the issue of overcrowding in advanced medical institutions and underutilization of resources in primary healthcare facilities, ultimately achieving a healthcare delivery model where patients with minor illnesses are treated in community-level institutions, serious illnesses are treated in general hospitals and recovery occurs in the community. Nevertheless, the increasing accessibility of outpatient settlement for cross-regional medical treatment would motivate a part of insured individuals to seek treatment at advanced medical institutions outside their registered residence. This trend could lead to a rise in outpatient visits to these advanced medical institutions, thereby hindering the implementation of the hierarchical medical system. However, research on this phenomenon remains limited.” (on lines 36-83 of the manuscript and lines 43-90 of the revised manuscript with track changes.)*

Again, we sincerely thank Dr. Matteo and the reviewers for your thoughtful and constructive comments on our manuscript, which have significantly contributed to its improvement. We hope that the revised manuscript meets the journal's requirements.

If you require any further information, please do not hesitate to contact us at slh-3632@163.com. We look forward to hearing from you.

Kind regards,

Lihua Sun, Prof.

Department of Pharmacy Administration, School of Business Administration, Shenyang Pharmaceutical University
